# Generation and Selection of Specific Aptamers Targeting *Brucella* Species through an Enhanced Cell-SELEX Methodology

**DOI:** 10.3390/ijms23116131

**Published:** 2022-05-30

**Authors:** Dalia M. El-Husseini, Ashraf E. Sayour, Falk Melzer, Magda F. Mohamed, Heinrich Neubauer, Reham H. Tammam

**Affiliations:** 1 Biotechnology Department, Animal Health Research Institute, Agricultural Research Center, Dokki, Giza 12618, Egypt; 2 Institute of Bacterial Infections and Zoonoses, Friedrich-Loeffler-Institut, 07743 Jena, Germany; heinrich.neubauer@fli.de; 3 Molecular Biomimetics Research Group, Animal Health Research Institute, Agricultural Research Center, Dokki, Giza 12618, Egypt; sayourashraf@gmail.com; 4 Chemistry Department, Faculty of Science, Cairo University, Giza 12613, Egypt; magdafikry85@yahoo.com (M.F.M.); reham_tammam@cu.edu.eg (R.H.T.)

**Keywords:** *Brucella*, aptamer, enhanced cell-SELEX, qPCR, high-throughput sequencing

## Abstract

Brucellae are Gram-negative, aerobic, non-motile coccobacilli causing brucellosis in man and animals. The disease is one of the most significant yet neglected global zoonoses. Especially in developing countries, brucellosis is causing public health problems and economic losses to private animal owners and national revenues. Composed of oligonucleotides, aptamers are chemical analogues of antibodies that are promising components for developing aptamer-based rapid, sensitive, and specific tests to identify the *Brucella* group of bacteria. For this purpose, aptamers were generated and selected by an enhanced protocol of cell systematic evolution of ligands by exponential enrichment (cell-SELEX). This enhanced cell-SELEX procedure involved the combination of both conventional and toggle cell-SELEX to boost the specificity and binding affinity to whole *Brucella* cells. This procedure, combined with high-throughput sequencing of the resulting aptamer pools, comprehensive bioinformatics analysis, and wet lab validation assays, led to the selection of a highly sensitive and specific aptamer for those *Brucella* species known to circulate in Egypt. The isolated candidate aptamer showed dissociation constant (K_D_) values of 43.5 ± 11, 61.5 ± 8, and 56 ± 10.8 nM for *B. melitensis*, *B. abortus*, and *B. suis*, respectively. This is the first development of a *Brucella*-specific aptamer using an enhanced combination of conventional and toggle cell-SELEX to the authors’ best knowledge.

## 1. Introduction

Brucellosis is caused by facultative intracellular bacteria of the genus *Brucella.* It is one of the major global bacterial zoonoses with a focus on the Mediterranean region and the Middle East [1,2,3]. Despite the efforts and progress that have been achieved in controlling the disease, it remains both a major threat to the health of livestock and humans and an economic burden.

*Brucella* species infections in both livestock and humans are caused by *Brucella melitensis*, *Brucella abortus*, and *Brucella suis,* which usually infect small ruminants, cattle, and pigs, respectively. Cross-transmission of *Brucella* species among animal species is possible [4]. Both horizontal and vertical transmissions are common among animals but not in humans [5,6,7]. Brucellae are associated with their animal hosts’ reproductive organs and lymph nodes and may be found in high numbers in urine, milk placental fluids, and aborted fetuses. Economic losses in animal production result from acute febrile illness, late abortion, weak offspring, an extensive decline in milk yield, and reduced fertility [8,9]. Vaccines can reduce the loss caused to the animal owner but will not protect against infection [10]. The chronically infected animal is the reservoir for new infections in herds and keeps the infection process active. Due to the intracellular lifestyle of brucellae, antibiotic therapy often fails and is thus prohibited in animals in many countries. Humans get infected via the consumption of unpasteurized milk or when handling infected animals or aborted fetuses. Undulant fever, night sweats, fatigue, arthralgia, and abscesses in all organs are unspecific symptoms. The disease turns chronic and relapses often occur after therapy has failed [11]. Consequently, preventing brucellosis in humans is strongly dependent on the containment of infected animals and monitoring their contaminated products [12].

A reliable and fast diagnosis of infectious diseases is the key to successful outbreak detection and disease spread prevention [13]. Currently available diagnostics include isolation of the causative agents as the gold standard and serological assays as the practical tools for massive testing. These techniques are generally time-consuming and need appropriate biosafety level (BSL) 3 laboratory setup, as well as trained personnel [14,15]. They bear the risk of contamination or false results [16]. As an alternative, molecular techniques such as polymerase chain reaction (PCR)-based assays have been explored to overcome the requirement of BSL-3 labs in case of culture and phenotypic identification [17]. Although PCR-based assays are safer for personnel, specialized instruments such as thermocyclers are needed along with expensive reagents and skilled personnel [18].

Direct detection of *Brucella* antigens has been hampered by the lack of sensitivity and/or specificity [19]. These limitations were manifested during either the detection of the whole-cell [20,21] or its subunits [22]. Nowadays, rapid diagnostics gain researchers’ attention, especially those involving aptamers as a detection agent [23]. Aptamer technology has opened the way for a new diagnostic branch and the developed tests are cheaper, faster, and more sensitive than some of the currently available methods [24]. This technology can be used together with optical [25], electrochemical [26], and mechanical [27] biosensors to eliminate some of the problems associated with traditional methods. Aptamers are used in many diagnostic methods, such as lateral flow [28] and fluorescence-based assays [29], appropriate for rapid field testing, especially in endemic areas.

Aptamers are short nucleic acids either single-stranded deoxyribonucleic acid (DNA) or ribonucleic acid (RNA) molecules. They usually range from 20 to 60 nucleotides that can fold into a unique three-dimensional (3D) conformation so that they can bind to their targets [30]. They can specifically bind to a wide range of ligand targets, from simple inorganic molecules [31] that antibodies cannot recognize to large protein complexes [32] and cells [33]. Aptamers are actually nucleotide analogues to antibodies with much more advantages. For instance, aptamer production is significantly easier and more cost-effective than antibodies, as they can be massively produced by chemical processes. They show high specificity and binding affinity to their targets equal to or even greater than antibodies [34] for direct target detection, especially for hard-to-cultivate bacteria like Brucellae. An additional advantage of aptamers over antibodies is that they may be chemically modified more easily, particularly for incorporating signal moieties like fluorophores and quenchers [35]. Contrary to antibodies, aptamers are more robust at higher temperatures, and their thermal denaturation is reversible [36,37,38,39,40]. The above-mentioned advantages make aptamers favorable alternatives to antibodies in disease diagnosis [41], detection of biomolecules [42,43], imaging [44,45], and therapeutic applications [38,46] because of their flexibility and tolerance to a wide range of assay conditions.

A specific aptamer can be generated by an in vitro process called “SELEX”, which stands for systematic evolution of ligands by exponential enrichment. SELEX is an iterative cycle of four main steps; co-incubation, removing unbound sequences, elution of bound sequences, and amplification, after which the selected aptamers are identified by sequencing [47]. The selection starts with incubating the target with a randomized ssDNA/RNA library flanked by a known sequence at both ends, which are primer sites. Library complexity increases linearly with library central region randomness. An aptamer library is expected to have around 10^14^ to 10^16^ unique sequences [48]. Some of these sequences bind to the target upon incubation, while the rest are washed away by subsequent washing steps. The bound sequences are then eluted from the target and subjected to amplification to generate enough product for the next round of co-incubation [49].

The monitoring of SELEX progression, determining aptamer enrichment, and ending of the SELEX cycles is traditionally achieved by flow cytometry. This method requires the introduction of a fluorophore to the aptamer pool through PCR amplification using a fluorescently labeled forward primer [50]. In addition, increasing fluorescence intensity only shows the enrichment of target bound aptamers, not allowing clear quantification of bound aptamers in contrast to other methods such as qPCR [51]. During the last decade, qPCR has gained more attention in monitoring the SELEX procedure due to the advantage of absolute quantification of cell-bound aptamers, as well as monitoring cycle by cycle enrichment and allowing the calculation of the molar ratio of cell-bound aptamers [52]. In addition, other methods have been validated and reviewed for monitoring SELEX, such as UV–Vis spectroscopy, ELONA, and EMSA [53]. Still, they were either incompatible with some SELEX variants or required post electrophoresis silver staining or biotin labelling and target immobilization, respectively, unlike qPCR, which was compatible with all SELEX variants and capable of providing sensitive SELEX evaluation.

SELEX was originally developed to select aptamers against a single protein, but with progress in technology and market requirements, various types of SELEX were established, including cell-SELEX [54,55]. Cell-SELEX technology possesses more advantages than traditional SELEX. For instance, it works on the protein in its native structure where conserved biological characteristics on the cell membrane are not affected by the protein purification procedure [56]. In addition, prior knowledge of certain outer-membrane proteins is not required [57]. In contrast, cell-SELEX may require more rounds for aptamer screening and the screening results can be affected if any damage occurs to target cells [56]. Cell-SELEX has been successfully employed to select aptamers against bacterial targets such as *Campylobacter jejuni* [58], *Vibrio parahemolyticus* [59], *Salmonella enteritidis* [60], *Streptococcus* [61], *Escherichia coli* [62], *Mycobacterium tuberculosis* [63], *Staphylococcus aureus* [64], and *Legionella pneumophila* [65].

Herein, we report the generation and selection of a highly sensitive and specific aptamer for direct broad-range detection of *B. melitensis*, *B. abortus*, and *B. suis* using an enhanced cell-SELEX technology altering between toggle and conventional cell-SELEX together with high-throughput sequencing for deeper analysis of the library and, hence, better aptamer selection.

## 2. Results

### 2.1. PCR Amplification and ssDNA Generation

We performed two PCR methodologies to amplify aptamer sequences. The results showed that the optimized conditions for the first method, direct asymmetric PCR, regarding annealing temperature, forward: reverse primer ratio, and number of cycles were 62 °C, 20:1 (500:25 nM), and 40 cycles, respectively (Figure S1). The optimized conditions for the second approach were as follows 62 °C and 8 cycles regarding annealing temperature and number of cycles, respectively, for symmetric PCR, while 62 °C and 20:1 (500:25 nM) regarding annealing temperature and forward to reverse primer ratio, respectively for asymmetric PCR (Figures S2 and S3).

### 2.2. qPCR Monitoring of Cell-SELEX and Enrichment Determination

**Figure 1:**
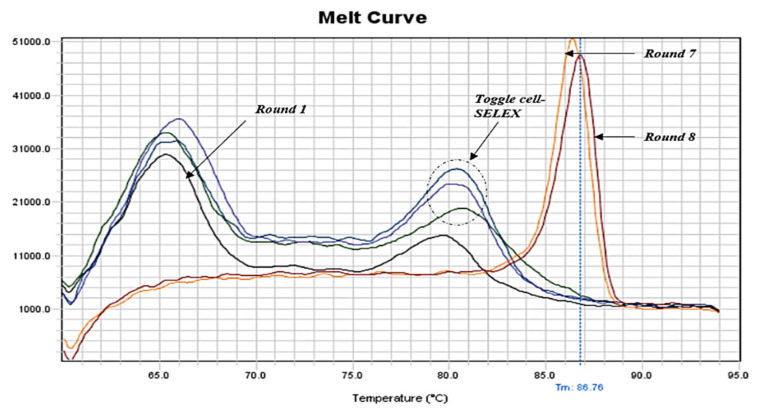
qPCR melting curves of positive cell-SELEX rounds indicating aptamer enrichment and ending of the cell-SELEX procedure.

The enrichment of aptamers throughout the cell-SELEX procedure was monitored by qPCR (SYBR Green method). The melting profiles showed that the pool’s diversity decreased gradually with the progress of SELEX. Moreover, after the toggle cycle (round 3–5) and the second negative selection, only one sharp peak continued to appear (rounds 7 and 8), indicating aptamer enrichment and the end of cell-SELEX cycles (Figure 1). Additionally, we noticed that the melting curve of the first round showed fewer artifacts than the initial aptamer library, indicating less heterogeneity and removal of some aptamer sequences from the pool (Figure S4a). Furthermore, the melting curves of the toggle cycle indicated enrichment of aptamers with *B. melitensis*, *B. abortus,* and *B. suis* (Figure S4b). The melting curve of the negative selection round six after the toggle cycle showed one sharp peak compared to round two (Figure S4c).

### 2.3. High-Throughput Sequencing Results, Bioinformatics Analysis, and Aptamer Selection

Prior to the high-throughput sequencing, an eluted aptamer pool of each cell-SELEX round was amplified using a special overhang primer set containing index sequences compatible with Illumina adaptor indexes resulting in a 162-bp PCR product (Figure S6).

All fastq files of the analyzed rounds showed good sequence quality with scores between 32–34 (Figure S7), with a gradual increase in GC percentages suggesting successful aptamer enrichment over SELEX rounds (Table S1).

**Figure 2:**
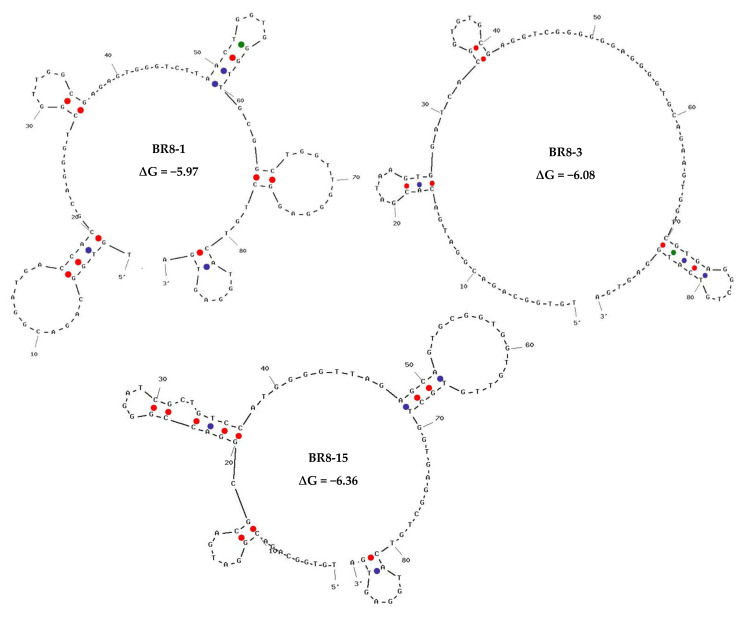
Secondary structure prediction and minimal free energy calculation for the selected aptamers using the UNAFold tool, IDT.

The top 100 sequences from each round, based on their frequency, were selected for further analysis and comparison. In case families were found, only one representative was selected. Out of these sequences, we first ranked the sequences according to their frequency in *Brucella* round 8 (BR8) and tracked them over the preceding positive rounds of SELEX via the fastaptamer_enrich tool. A list of the top 10 most frequent sequences that showed good enrichment was created (Table S2). The following criteria were used for selecting sequences, *viz*. sequence frequency, early rounds appearance and showing enrichment throughout SELEX rounds. The selected sequences were then compared to sequences of non-target cell-bound aptamers in the counter selection round. As a proof of concept, we chose three sequences that showed good enrichment in cell-SELEX with high and low sequence read in non-target cell-bound aptamers to determine the importance of negative selection round and efficiency of sequence analysis (Table 1). The selected sequences were subjected to secondary structure prediction and minimal free energy calculation using the UNAFold tool, IDT (Figure 2).

### 2.4. Specificity, Binding Affinity, and Sensitivity of Selected Aptamers

**Figure 3:**
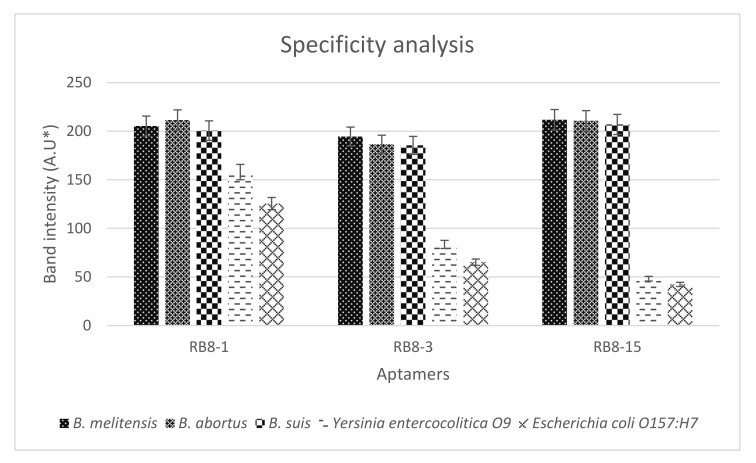
Specificity analysis of RB8-1, RB8-3, and RB8-15 for *B. melitensis, B. abortus, B. suis, Yersinia entercocolitica* O9, and *Escherichia coli* O157:H7. * A.U = arbitrary unit.

**Figure 4:**
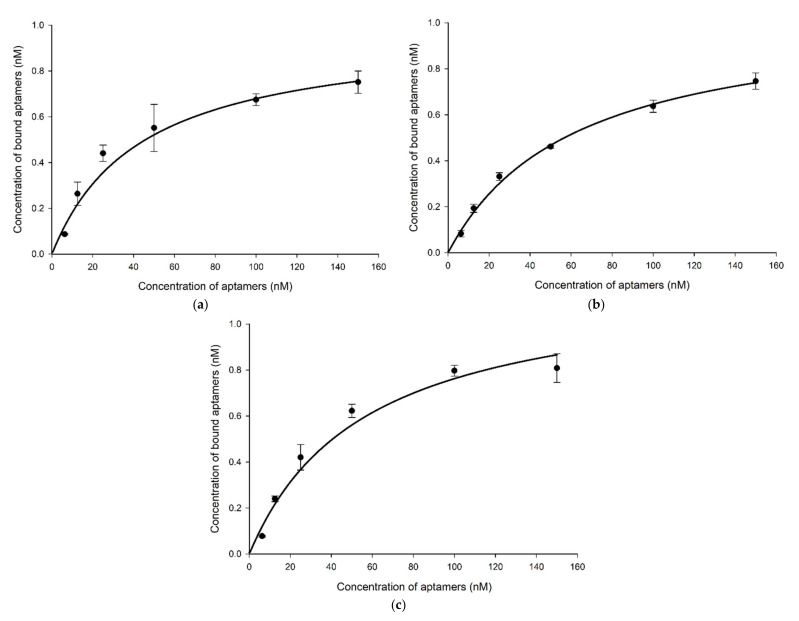
Binding affinity of aptamer BR8-15 with (**a**) *B. melitensis* K_D_ = 43.5 ± 11 nM, (**b**) *B. abortus* K_D_ = 61.5 ± 8 nM, and (**c**) *B. Suis* K_D_ = 56 ± 10.8 nM.

The three synthesized aptamers were first tested for their specificity to *Brucella* species and other bacteria in counter selection rounds by PCR to rule out the nonspecific aptamers before testing their sensitivity and predicting their binding affinity. The results indicated that all aptamer candidates exhibited a similar level of binding towards all *Brucella* species. Not to mention, all aptamers displayed higher binding for the target cells than for the non-target cells (Figure 3 and Figure S8). However, gel band intensities for BR8-1 showed closer measurements between target and non-target cells. BR8-3 band intensities for non-target cells had lower measurements than that of BR8-1. BR8-15 band intensities for target cells showed comparable measurements to BR8-1 and BR8-3. Nevertheless, it showed the lowest band intensity measurements for non-target cells. BR8-15 presented a better difference in band intensity measurements between target and non-target cells than BR8-1 and BR8-3. Based on the specificity results, the BR8-15 aptamer was selected to further analyze its sensitivity and binding affinity. BR8-15 showed a limit of detection of 10^1^ CFU/mL for each of the *Brucella* species (Figure S10). The determined dissociation constant (K_D_) values for the three *Brucella* species were between 30 and 70 nM, indicating a high binding affinity between the BR8-15 aptamer and its targets. The K_D_ values were 43.5 ± 11, 61.5 ± 8, and 56 ± 10.8 nM for *B. melitensis*, *B. abortus*, and *B. suis*, respectively (Figure 4 and Figure S9). These values were comparable to other K_D_ values previously reported for aptamers bound bacterial cells, usually in the range of 10–200 nM [52,66,67].

## 3. Discussion

Throughout the years, SELEX has been improved in terms of the selection procedure and automation [66,67]. These improved methods provide fast results; however, most of these methods require costly reagents and equipment often unavailable in ordinary laboratories [68]. The conventional SELEX procedure is still widely used as a preferred method for aptamer selection, especially for large targets such as bacterial cells. Nevertheless, it suffers from several pitfalls [69], viz., (1) PCR amplification artifacts and bias due to the random nature of the aptamer library, (2) the generation of high quality and enough quantity for ssDNA at the start of each round, and (3) the requirement of many SELEX rounds for successful aptamer enrichment to obtain high-affinity aptamers, which is time and reagent consuming. Typically, an average of 12 to >20 positive SELEX rounds with a two- to ten-month performance time scale is required to obtain high-affinity aptamers. This period depends on several factors such as bacterial target nature, whether immobilized or not, partitioning methods, and ssDNA generation method [70]. In this study, we designed an improved cell-SELEX procedure associated with high-throughput sequencing and intensive in silico analysis in order to avoid common SELEX pitfalls.

The designed cell-SELEX procedure aimed to combine the cell-SELEX conventional method where one strain was only used or a mixture of different strains of the same species was pooled in one tube [61] with the toggle cell-SELEX that was used to select broadly reactive aptamers for different species [61]. Adding one cycle of toggle cell-SELEX ensured the binding of aptamers to all strains and avoided their bias to a certain dominant strain which would later be reflected in the binding affinity [61,71]. Additionally, increasing the selection pressure as the cell-SELEX procedure progressed resulted in attaining the aptamer enrichment after only a few cell-SELEX rounds, usually achieved after twelve or more positive selection rounds [72]. We added extra components such as tRNA and BSA to the reaction buffer [73]. tRNA was added to the binding buffer to compete with the aptamer for the binding sites of the bacterial outer membrane [74]. Likewise, BSA was added to the binding buffer to compete with the bacteria for binding with aptamer and it was added to the washing buffer to aid in the removal of nonspecifically bound aptamers on the bacterial surface [59]. In the first round, less harsh conditions were applied to allow aptamers to bind to the bacterial outer membrane and avoid the loss of aptamer sequences that could be specific due to the low starting copy number of each unique aptamer sequence. Afterwards, the stringency of the conditions was increased gradually to allow only the specific strongly bound aptamers to be upheld with the progress of the cell-SELEX procedure [72]. Moreover, two rounds of negative selection, not only one round as in conventional cell-SELEX, were carried out to remove the aptamer sequences that could bind to both targeted bacteria and other bacteria with similar outer membrane structures and boost the specificity. The presence of mixed cell types in the incubation step might as well serve as competitive binders for each other, increasing the selection stringency [75]. The subsequent screening of the selected aptamers was carried out to identify the specificity of those aptamers to each *Brucella* strain.

There are several methods for separating target-bound aptamers and unbound aptamers [68]. The easiest and most convenient way is centrifugation which depends on the mass of cells that will be pelleted, leaving the unbound aptamers in the supernatant [76]. The washing step then removes the weakly bound aptamers. After washing, heating the pelleted cells to 95 °C elutes the bound sequences. This high temperature denatures the cell outer membrane proteins leading to the disruption of aptamer and protein interaction allowing the release of aptamers into the supernatant. Additionally, the elevated temperature ensures the inactivation of any DNase released after cell disruption preventing aptamer digestion [57].

In PCR amplification, we used a high-fidelity enzyme and a GC enhancer to preserve aptamer sequence and avoid PCR bias, respectively. After each cell-SELEX round, the eluted aptamers were amplified by PCR, as these molecules were present in very minute amounts, insufficient to perform the next round. PCR amplification optimization in the SELEX experiment was vital for the success of subsequent procedures. Herein, we experimented with two methods: the first was to simultaneously amplify aptamer pools and generate ssDNA using asymmetric PCR, and the second was to first amplify the aptamer pool by symmetric PCR and use its product to generate ssDNA by asymmetric PCR. Both methods showed good amplification in early rounds and produced adequate ssDNA for the following round. However, with the progress in cell-SELEX rounds, the first method showed more and more byproducts affecting the ssDNA formation and concentration. This observation might be due to the forward primer partial binding to complementary bases in the N-region of aptamer sequences followed by the extension of this region by asymmetric PCR or partial binding of asymmetric PCR products or primers to the denatured bacterial contents. These by-product phenomena have been extensively discussed by Tolle et al. [77]. In contrast, the second method kept providing good amplification and adequate ssDNA generation. This could be explained as the application of symmetric PCR prior to asymmetric PCR provided higher template concentration and more purified bacterial contents, especially in the later rounds. These observations led us to eventually stop using the first method after the fifth cycle and continue only with the second one. We also noticed that the cycle number for symmetric PCR was altered in the range of 6 to 12 cycles according to the cell-SELEX round number. We suggest that due to the unpredictable continuous change of bacterially bound aptamer concentration with each SELEX round, the PCR cycle number should be optimized for each cell-SELEX round before performing the large-scale asymmetric PCR of 200 μL. This way ensures the production of enough templates with no non-specific products for the subsequent asymmetric PCR. Asymmetric PCR scale-up was performed in 8 patches of 25 µL to obtain 200 µL of PCR product which was enough to give approximately 200 pmol of ssDNA to be used in the co-incubation step of the successive cell-SELEX round. Usually, 1 mL of PCR product was required to produce enough ssDNA after the loss occurred in the ssDNA generation step using other procedures [57,78]. We used less PCR reaction volume due to the high concentration of the generated ssDNA using combined symmetric and asymmetric PCR in addition to the high yield production of the ssDNA purification method that we used.

Most studies proved the validity of performing one-step asymmetric PCR for high-quality generation of ssDNA for SELEX using either the original aptamer library or the produced aptamer pool after the first cycle [79,80]. This agrees with our observations for the early few cycles of SELEX. Yet, our observations were in contrast to other publications that apply asymmetric PCR to different aptamer pools of several SELEX rounds. They indicated that with the progress of SELEX rounds, the ratio of ssDNA to dsDNA increased due to the more homogeneity of aptamers [81]. However, this observation may be due to the absence of bacterial genomic and transcript content, as it was not mentioned in the method generating the aptamer pool [81]. Other studies indicated that even though with asymmetric PCR optimization, non-specific products could affect the aptamer sequence preservation and give rise to PCR artifacts [79].

Successful generation of ssDNA from the amplified dsDNA is one of the critical steps in the SELEX procedure essential for forming aptamer 3D structures required for target binding, whereas dsDNA only had the double helix conformation. Various methods are usually used for this step, most commonly, biotin-streptavidin separation. Still, it requires very expensive reagents, and sometimes, the undesired biotinylated ssDNA can escape to the eluted desired ssDNA leading to re-annealing to its complementary strands resulting in the loss of aptamers’ tertiary structure [82]. Another method, lambda exonuclease digestion, has the limitation of incomplete enzymatic digestion, 5′ phosphorylation of one of the primers and requirement of subsequent purification [83]. Other novel and combinatorial methods were used and evaluated [84,85]. Asymmetric PCR is an alternative method performed with an unequal molar ratio of forward and reverse primers where the reverse primer (usually the lower concentration primer) is incorporated into dsDNA, while the forward primer is used to produce excess ssDNA in each cycle. At the end of PCR amplification, the amplified product usually comprises a mixture of ssDNA and dsDNA, which needs to be separated on either non-denaturing PAGE (preferred) or high percentage agarose gel, followed by extraction and purification of the required product from the gel [80]. Most gel extraction kits are mainly used for dsDNA purification and/or larger strands, leading to a poor yield of ssDNA aptamer sequences. Thus, this method was considered time-consuming [84]. Herein, we considered a fast and reliable ssDNA/RNA cleaner and concentrator^TM^ kit that can be performed in 10 min, works directly on PCR product, eliminates dsDNA, eliminates very short strands (primers), and purify and concentrate ssDNA in the range of >17–200 (designed aptamer; 90 b). The resulting purified aptamers from this kit showed high purity (A260/A280 ratio) and high recovery.

Efficient monitoring of the SELEX progression is crucial for successfully determining aptamer enrichment and SELEX ending. Herein, we applied qPCR amplification followed by a standard melting procedure which resulted in the plotting of melting curves. Comparison of melting curves of aptamer pools from each cell-SELEX round provided further evidence of aptamer enrichment throughout the designed cell-SELEX procedure and gave an indication of when SELEX should end. Our melting curves showed multiple broad peaks for the initial aptamer library and early rounds, while they tended to show one sharp peak as rounds progressed. Melting curves of rounds seven and eight showed comparable one sharp peak indicating the end of the designed cell-SELEX rounds. Since the melting temperature depends on the structure of internal bases, the formed hetero- and homo-duplexes were presented by more than one peak due to the high sequence diversity of aptamers. However, with the progress of cell-SELEX rounds, the hetero-duplex peak (often at a Tm of 70–72 °C) should decrease while the homo-duplex peak (often at a Tm of 82–87 °C) should increase. Upon approaching the end of SELEX, the amplification plot and melting curves typically reached a stable state [86]. Therefore, the initial aptamer library and early aptamer pools were expected to form multiple and/or broad peaks due to the factor of aptamer sequence diversity. With the progress of the cell-SELEX procedure, the aptamer pool diversity decreased and consequently, the peaks became sharper and uniform [87]. Our results were in concordance with another publication that used the same methodology in monitoring SELEX progress [53]. qPCR proved to be an efficient monitoring tool and effective alternative with fewer requirements, low cost, and time-saving advantages. All our findings from melting profiles analysis were later supported by HTS results.

Another major concern in SELEX was the correct determination of aptamer sequences, frequency of each sequence in the pool, and diversity of the pool. Since the frequency of all aptamers, even if from the enriched SELEX round, was not monotonic, then theoretically, it should be represented in the results of conventional cloning and sequencing techniques, but experimentally bacterial colonies sometimes do not essentially contain different aptamer sequences. This could be due to PCR bias and the dominance of some aptamer sequences over others. Hence, this approach was considered time-consuming because it limited the number of sequences to be determined and provided inaccurate aptamer sequence frequency estimation. Recent advances in high-throughput sequencing applications opened a new avenue to a more accurate post-selection examination of aptamer pools in terms of determining aptamer sequences, frequency, diversity, and enrichment [88]. The principal objective of the high-throughput SELEX analysis approach was to inform the presence of target-specific candidate aptamers before downstream application and experimental validation took place. High-throughput sequencing was applied in our study to every SELEX round to determine aptamer sequences while simultaneously tracking the enrichment trajectory of each aptamer sequence.

Data analysis of high-throughput sequencing showed sequence enrichment starting from cell-SELEX round BR3, where more than 10 reads/sequence appeared. Starting from BR6 till BR8, more than 100 reads/sequence up to 4000 reads/sequence showed up, indicating high sequence enrichment. This observation agrees with our qPCR findings, where the melting curve showed one narrow peak from BR6 and beyond. We also observed a few aptamer sequences which appeared in BR3 and BR4 and then disappeared in the following rounds, while others continued in the following rounds but in low count. This sequence appearance could be due to the possibility of PCR artifacts and bias [88]. We also observed that the top 20 sequences in BR7-BR8 almost kept their ranking with an increase in their frequency. An analysis of aptamer sequences of different rounds demonstrated the evolution of sequences inside each round population, revealing that the most frequent aptamer sequences in the final round were not always the best choice in terms of target specificity or binding affinity. These findings agree with other publications using the same technology with different data analysis methods [89,90,91].

From our analysis, using the FASTAptamer toolkit, based on the criteria of sequence ranking, showing enrichment throughout SELEX rounds, especially the last two positive rounds and subtraction of enriched sequences of positive rounds from enriched sequences in the negative selection round, we deduced that the most promising aptamer would be “BR8-15”. BR8-1, BR8-3, and BR8-15 aptamers were enriched throughout the cell-SELEX rounds, especially round numbers seven and eight and were present in the aptamer pool of early rounds. However, BR8-1 and BR8-3 also showed somehow high sequence frequency in the non-target aptamer pool (negative selection) and were ranked in the third and the twelfth places in round six. On the contrary, BR8-15 showed low frequency and was ranked sequence No. 5145 in round six. Accordingly, we assumed that BR8-1 and BR8-3 would show some binding to non-target bacteria rather than BR8-15, which was confirmed later in the wet lab analysis. Hence, we recommend that if high-throughput sequencing is used, it is better to analyze the sequences of the last two positive selection rounds against the sequences of the last negative selection round and perform intensive bioinformatics analysis before selecting aptamers for laboratory validation.

Overall, our procedure from aptamer library design to the selection of candidate aptamer showed great potential in the aptamer generation field. However, further studies would be needed to identify the *Brucella* cell-surface epitope to which the aptamer has an affinity. Future comprehensive studies regarding testing the aptamer sensitivity, specificity, and binding affinity in real samples with complex matrices would be necessary to guarantee the efficiency and robustness of the selected aptamer if going to be used in diagnostic applications. Furthermore, other targets should be experimented with to prove the reproducibility of our designed cell-SELEX method, as well as the relation between qPCR melting curves and high-throughput sequencing result analysis.

## 4. Materials and Methods

### 4.1. Bacterial Targets, Culturing Conditions, and Dilution

*Brucella abortus* biovar 1 strain 544 (ATCC no. 23448, The American Type Culture Collection, Manassas, VA, USA), *B. abortus* biovar 1 strain 19 (vaccine strain), *B. melitensis* biovar 1 strain 16 M (ATCC no. 23456), *B. melitensis* biovar 3 strain Ether (ATCC no. 23458), *B. melitensis* biovar 1 strain Rev1 (vaccine strain), and *B. suis* biovar 1 strain 1330 (ATCC no.1330) were used for the positive cell-SELEX rounds. *Yersinia entercocolitica* O9 and *Escherichia coli* O157:H7 were used for the negative cell-SELEX rounds. Bacterial culture activation by propagation on a solid medium was performed according to Alton et al. [92] as for vaccine production.

Briefly, all *Brucella* strains were cultured on Brucella agar, Difco Z.B for 72 h at 37 °C with 5% CO_2_. The other Gram-negative bacteria were grown onto tryptone soy agar plates at 37 °C for 24 h. Colonies were picked and sub-cultured on agar plates under the same conditions to activate bacterial cultures before every cell-SELEX round. Different cell densities ranging from 10^5^ to 10^8^ of the fresh bacterial cultures were prepared for each SELEX round by matching McFarland turbidity standards and measuring their concentration at OD_600_.

### 4.2. ssDNA Library and Primers Design

To obtain a more complex library, the ssDNA library was designed to have 55 Ns with a ratio of 25:25:25:25 for A:T:G:C and flanked by constant regions at both ends acting as primer sites. The constant region sequences were designed to avoid any secondary structure formation (hairpins, homodimer, and heterodimer). The designed ssDNA library used in this study is “TGT GGC AGA CGG ATG AC (N55) GAG GCT GTC ATG GAG TGA”, and for PCR amplification, the following primer set was used; forward primer: TGT GGC AGA CGG ATG AC and reverse primer: TCA CTC CAT GAC AGC CTC. The library and primers were HPLC purified and obtained from (IDT Inc., Coralville, IA, USA) in the lyophilized form. They were rehydrated as recommended by the producer’s instructions to obtain a concentration of 100 µM.

### 4.3. Cell-SELEX Workflow

**Figure 5:**
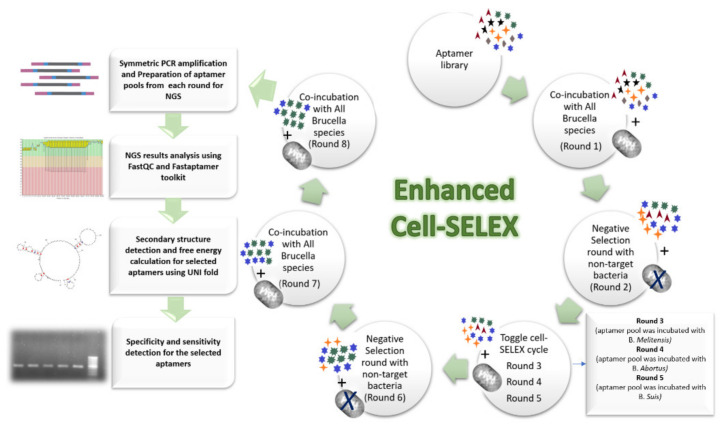
Schematic representation of the designed enhanced Cell-SELEX steps from initial aptamer pool until the selection of efficient aptamer sequence.

In this work, the enhanced cell-SELEX procedure was designed based on integrating two types of cell-SELEX, namely conventional and toggle cell-SELEX. This combination aims to enhance target specificity and decrease the number of required rounds for attaining aptamer enrichment. In total, three conventional cell-SELEX rounds, two negative selections; and one toggle cell-SELEX were performed to select a specific aptamer for the most common *Brucella* species (*B. abortus*, *B. melitensis*, and *B. suis*). Between rounds, bounded aptamers were separated, amplified, and used for ssDNA generation in sufficient quantity required for next round co-incubation. The ending of cell-SELEX rounds was determined by monitoring the progress of each round by qPCR until the melt curves of two successive rounds showed comparable results. Sequences of aptamer pools were then identified by HTS, followed by candidate sequences selection and wet-lab validation (Figure 5).

In conventional cell-SELEX, all *Brucella* strains were pooled in one tube and allowed to interact with the aptamer pool. Wherein, toggle cell-SELEX, each *Brucella* species was allowed to interact with the aptamer pool then the resultant amplified eluted aptamers were then incubated with another *Brucella* species, and so on. The negative selection round was similar to conventional cell-SELEX but with incubating the aptamer pool with other bacterial cells with similar outer membrane structures to the target cells.

### 4.4. Cell-SELEX Rounds

Before each SELEX round, freshly prepared binding and washing buffers were used. The binding buffer was composed of 1X PBS buffer (pH 7.0), bovine serum albumin (BSA) 1–60 mg/mL (according to SELEX cycle number), yeast tRNA 0.1–0.4 mg/mL (according to SELEX cycle number), and ssDNA library (1 nmol for the first round and approximately 200 pmol for the successive rounds). The washing buffer consisted of 1X PBS and 0.05% BSA.

The ssDNA library was denatured at 95 °C for 10 min and immediately cooled on ice for 10 min before the co-incubation started. The denatured library was mixed with the bacterial cells and co-incubated in the binding buffer at room temperature for 45 min under shaking at 200 rpm. With the progress of the cell-SELEX rounds, selection pressure was increased. The incubation period gradually decreased from 45 to 15 min. The shaking speed increased gradually from 200 to 800 rpm. Bacterial copy number decreased gradually from 10^8^ to 10^5^, and the concentration of BSA and tRNA increased to 60 mg/mL and 0.4 mg/mL, respectively. Likewise, the washing volume (250–1000 µL), incubation time (1–5 min), and frequency (1–3 times) were increased (Table 2).

Generally, before each round, the harvested bacterial cells regarding cell-SELEX round number were centrifuged at 10,000 rpm for 10 min, followed by washing the pellets with 1X PBS, pH 7.0. For the conventional positive cell-SELEX round, an equal volume of each of the seven diluted *Brucella* strains was pooled into a 1.5-mL Eppendorf tube. Whereas each *Brucella* species was used separately in the toggle cell-SELEX round, the *Brucella* strains of the same species were pooled together into a 1.5-mL Eppendorf tube and used according to the toggle cycle. For negative selection rounds, an equal volume of diluted 10^7^ to 10^8^ CFU/mL of *Yersinia entercocolitica* O9 and *Escherichia coli* O157:H7 was pooled together.

For positive selection rounds, after co-incubation, the mixture was centrifuged at 10,000× *g* for 10 min for precipitation of aptamer–target complex, leaving the unbound aptamers in the supernatant, which was discarded, followed by washing the pellet with washing buffer. The pellet was resuspended in 30 µL of DNase/RNase-free water. The bound aptamers were eluted from the complex by heating at 95 °C for 10 min. The mixture was then centrifuged, and the supernatant containing the eluted aptamers was transferred into a new 1.5-mL Eppendorf tube. For counter/negative selection rounds, after co-incubation, the mixture was also centrifuged at 10,000× *g* for 10 min, followed by the transfer of the supernatant containing the unbound aptamers into a new 1.5-mL Eppendorf tube to be purified and concentrated using ssDNA/RNA clean and concentrator^TM^ kit (Zymo Research Co., Irvine, CA, USA, Cat. No. D7010).

### 4.5. PCR Amplification and ssDNA Generation

At the end of each cell-SELEX round, the eluted aptamers were subjected to PCR. In this study, we experimented with two methods.

#### 4.5.1. Asymmetric PCR Amplification

Reaction optimization in terms of annealing temperature, the ratio of forward to reverse primer concentration, and cycle number was performed to avoid PCR bias. Ranges of forward to reverse primer ratios from 10:1 to 100:1 were experimented. Different cycle numbers ranged from 20 to 50 cycles, as well as different annealing temperatures from 58 to 66 °C were applied.

#### 4.5.2. Symmetric Followed by Asymmetric PCR Amplification

For symmetric PCR, reaction optimization in terms of annealing temperature (58–66 °C) and number of cycles (6–15 cycles) were carried out. Two microliters of the symmetric PCR product were then added to the asymmetric PCR mixture for reaction optimization in terms of annealing temperature (58–66 °C) and forward to reverse primer concentration ratio (10:1 to 100:1).

For both methods, optimizations were performed using Q5^®^ High-Fidelity DNA Polymerase (NEB #M0491) in a 25 μL reaction volume containing 0.5 μL of 10 mM dNTPs, 5 μL of 5X Q5 reaction buffer, 5 μL of 5X Q5 high GC enhancer and 0.25 μL Q5 High-Fidelity DNA Polymerase. Forward and reverse primers were added according to the corresponding validation step. PCR program was applied as follows; 1 cycle of initial denaturation at 98 °C for 30 s and 6–50 cycles of denaturation at 98 °C for 5 s, annealing at 58 to 66 °C for 10 s, and extension at 72 °C for 10 s. A final hold at 4 °C was applied until the PCR product was either migrated on gel electrophoresis or subjected to the subsequent ssDNA purification. The annealing temperature was calculated according to New England BioLabs Tm Calculator, version 1.13.0 (http://tmcalculator.neb.com/#!/main (accessed on 30 January 2019). The deduced annealing temperature was 65 °C.

PCR optimization was performed on the eluted aptamers from the first cell-SELEX round and was subjected to modification as required with the progress of SELEX cycles. To produce enough ssDNA as an input for the next round (~200 pmol), 200 μL of PCR mixture was prepared for each round.

Asymmetric PCR product usually produces a few dsDNA copies beside ssDNA; therefore, the generated ssDNA products were extracted and purified by ssDNA/RNA clean and concentrator^TM^ kit (Zymo Research Corp., Irvine, CA, USA, Cat. No. D7010). The procedure was carried out as indicated by the manufacturer’s instructions with minor modifications. The elution step was performed twice using eight μL of DNase/RNase-free water and two min incubation time for each to ensure the elution of all attached ssDNA. The purified ssDNA concentration and purity were measured by UV/Vis nano spectrophotometer.

### 4.6. Gel Electrophoresis and ImageJ Analysis

After each optimization condition, all PCR products (5 μL each) were analyzed on 2.5% agarose gel electrophoresis for 30 min at 120 V. PCR products were visualized by a Gel-Doc device. ImageJ software was used to analyze and quantify PCR products band intensities of gel documented images [93]. The band intensities were then compared together to select the optimal conditions.

### 4.7. Monitoring of Cell-SELEX Progress and Aptamer Enrichment Using qPCR

Real-time PCR (qPCR) was used to monitor the progress in cell-SELEX rounds and the detection of aptamer enrichment. The qPCR mixture was prepared using the SensiFAST SYBR^®^ mix (2X) kit (Bioline, London, UK, Cat. No. BIO-94005). This analysis was performed in a 20 μL reaction composed of 10 μL of 2X SensiFAST mix, 0.8 μL of each primer, and ~4 ng/μL of template DNA (elution of each cell-SELEX round). qPCR thermal profile consisted of 2 min of initial denaturation at 95 °C, followed by 40 cycles of denaturation at 95 °C for 5 s, annealing at 60 °C for 10 s, and elongation at 72 °C for 10 s. After amplification, a melt-profile analysis was performed according to the StepOne thermocycler (Applied Biosystems, Waltham, MA, USA) instructions.

### 4.8. High-Throughput Sequencing (HTS) by Illumina Technology

After performing all cell-SELEX rounds, aptamer pools from each round were subjected to Illumina high-throughput sequencing to identify their sequence composition. Sample preparation and sequencing were performed in the Institute of Bacterial Infections and Zoonoses (IBIZ), Friedrich-Loeffler-Institut (FLI), Jena, Germany. Briefly, the eluted aptamers were first amplified using an overhang primer set (Metabion, Germany) compatible with Illumina adapter-indexes; forward-overhang: TCGTCGGCAGCGTCAGATGTGTATAAGAGACAGTGTGGCAGACGGATGAC and reverse-overhang: GTCTCGTGGGCTCGGAGATGTGTATAAGAGACAGTCACTCCATGACAGCCTC.

A Q5^®^ High-Fidelity DNA Polymerase kit was used for the symmetric PCR amplification of a total volume of 25 μL as follows; 5 μL of reaction buffer (5X), 0.5 μL of dNTPs (10 mM), 1 μL of DMSO, 0.5 μL of each primer (10 μM), 0.25 μL of Q5 High-Fidelity DNA Polymerase, and 10 ng of eluted aptamers from each round. Due to the required high annealing temperature, primer annealing, and extension were performed in one step as follows; initial denaturation at 98 °C for 30 s, denaturation at 98 °C for 5 s, and annealing/extension at 72 °C for 30 s. PCR cycle number was optimized for each round of eluted aptamers. After amplification, the PCR product was migrated on 2% gel at 100 V for 30 min. The specific bands at 162 bp were excised and subjected to DNA extraction and purification by QIAquick Gel Extraction Kit (Qiagen, Hilden, Germany, Cat. No.28704) according to the instruction manual. The concentration and purity of the purified PCR products were measured by Qubit^®^ 3.0 Fluorometer (Thermo Fisher Scientific, Waltham, MA, USA) using Qubit^®^ dsDNA HS Assay Kit (cat. No. Q32851).

Nextera™ XT Library Prep Kit (Illumina Inc., San Diego, CA, USA, lot No. 20286282, 2 boxes) and Nextera™ XT index Kit (Illumina Inc. lot No. 20235675) were used for library preparation as recommended by the manufacturer with minor modification, where the tagmentation step was replaced by the symmetric PCR amplification mentioned above. Briefly, ~1.5 ng of each purified PCR product representing the cell-SELEX rounds was further amplified using NPM (Nextera PCR Master Mix) to introduce the index sequences to aptamers. Limited PCR cycle conditions consisted of activation at 72 °C for 3 min, initial denaturation at 95 °C for 30 s, and 12 cycles of 95 °C for 10 s, 55 °C for 30 s, 72 °C for 30 s, and 72 °C for 5 min. AMPure XP beads were used according to Illumina instructions to purify the PCR products and remove short fragments and primers. The prepared libraries were analyzed and checked for uniformity and availability using Agilent High Sensitivity (HS) DNA kit (Life Technologies Co., Carlsbad, CA, USA, cat. No. 5067-4626) compatible with the 2100 Bioanalyzer instrument (Agilent, Santa Clara, CA, USA). Bead-based library normalization was performed according to the instructions manual to ensure equality of each library representation in the final pooled library. Afterwards, the prepared libraries were pooled together by pipetting equal volumes of each library into a new tube. The pooled libraries were diluted, denatured, and mixed with a positive control before it was loaded into the HTS cartridges using MiSeq Reagent Kit v2 (Illumina Inc., San Diego, CA, USA, lot. No. 20345005, 2 boxes) and Phix control V3 (lot. No. 20168446) as recommended by the manufacturer instructions. Miseq system (Illumina Inc., San Diego, CA, USA) was used for performing sequencing, and MiSeq Reporter software (MSR) provided analyses for MiSeq sequencing data regarding image analysis and base calling.

### 4.9. Bioinformatics Analysis and Aptamer Selection

FastQC (V.0.11.8) was used to analyze fastq files generated from the sequencer in terms of total sequences, GC content, per base sequence quality, per sequence quality scores, per base N content, and sequence length distribution and to give a hint of the overexpressed sequences and adapter contamination [94].

FastAptamer toolkit (V.1.0.3) was used for the primary sequence analysis of fastq files of the cell-SELEX rounds. It performed several tasks such as ranking, counting, normalizing, and sorting each of the abundant unique sequences in a given SELEX round population through the fastaptamer_count tool. Other tasks such as sequence fold enrichment, generating families, and comparing rounds could be achieved through fastaptamer_enrich, fastaptamer_cluster, and fastaptamer_compare tools, respectively. The fasta output file fastaptamer_count tool was used as an input for fastaptamer_cluster to generate sequence clusters based on Levenshtein edit distance of k = 7 [95].

The selected sequences were further subjected to secondary structure analysis and minimal free energy (∆G) calculation by the UNAFold tool, IDT (https://eu.idtdna.com/UNAFold (accessed on 5 March 2020). A workflow was designed to simplify the analysis of high-throughput sequencing results (Figure S5).

### 4.10. Specificity and Sensitivity Analysis of Selected Aptamers

Regarding specificity analysis, 10^6^ CFU/mL of freshly cultured target and non-target bacterial species were collected at the logarithmic phase and co-incubated with 100 nM of each aptamer candidate separately in 200 µL of binding buffer with shaking at 300 rpm for 15 min. The bacterial-bound aptamers were separated by centrifugation at 10,000 rpm for 10 min. The resultant pellet was washed twice with washing buffer, resuspended in 30 µL DNase/RNase-free water, and denatured at 95 °C for 10 min. Two microliters were then applied to symmetric PCR amplification for 20 cycles. PCR products were migrated on 2.5% agarose gel for 30 min at 120 V. Detected PCR product band intensities were analyzed using ImageJ software.

Likewise, for sensitivity analysis, a range of 10^0^–10^8^ CFU/mL of each target bacterial species was co-incubated with 100 nM of the selected aptamer (BR8-15). The following steps and PCR amplification were the same as specificity. All experiments were conducted in triplicates.

### 4.11. Binding Affinity Analysis of the Selected Aptamer

For binding affinity analysis, 10^7^ CFU/mL of each target bacterial species were co-incubated with different dilutions of BR8-15 aptamer ranging from 6.25–150 nM in 200 µL of binding buffer with shaking at 300 rpm for 15 min. After incubation, the mixture was centrifuged at 10,000 rpm for 10 min. The resultant pellet was then washed with washing buffer and resuspended in 30 µL of DNase/RNase-free water. The mixture was heated at 95 °C for 10 min to elute the bound aptamer sequences. The quantification of aptamers bound to the *Brucella* cells was carried out by qPCR analysis [52,96,97,98] (StepOne, Applied Biosystems) using SensiFAST SYBR^®^ mix (2X) kit (Bioline, Cat. No. BIO-94005). A ten μL qPCR reaction mixture was prepared containing 1 μL eluted bound aptamers, 5 μL of 2x SYBR mix, 0.2 μL of 10 μM forward primer, 0.2 μL of 10 μM reverse primer, and up to 10 μL of nuclease-free water. These reactions were carried out in triplicates. The cycling conditions followed the three-step cycling recommended by the manufacture with annealing at 58 °C. The resultant data were analyzed by StepOne^TM^ software v2.3. A standard curve (concentration ranging from 10^−3^ to 10^2^ nM of BR-15 aptamer) was constructed and used for bound aptamer quantification. The data were normalized against the negative controls and plotted to draw the saturation curves. The equilibrium dissociation constant (K_D_) was calculated by a non-linear regression curve fitting analysis using one site saturation equation (Equation (1)) of SigmaPlot software (v14.0), where Y is the quantity of cell-bounded aptamers, X is aptamer concentration, and $B_{max}$ = the specific aptamer maximum binding capacity.

(1) $$Y=B_{max}\frac{X}{K_{D}+X}$$

## 5. Conclusions

Aptamer-based assays are great potential tools in bacterial surveillance and diagnosis. Herein, we designed an enhanced, cost-, and time-effective cell-SELEX procedure to generate specific aptamers that can bind to *Brucella* species. The applied procedure involved several strategies to avoid SELEX tricks, such as (1) using high-sequence diversity in the initial pool (50 N) resulted in more possible sequences for binding and overall high affinity in their binding targets, (2) incorporating two counter selections using non-targets of similar outer membrane structure to the targets ensured high specificity, (3) combining two cell-SELEX types (conventional and toggle cell-SELEX) to avoid bias to a certain cell type in the mixture and ensured binding of aptamers to all selected cell types with comparable affinity, and (4) using high stringency conditions that gradually increase with the progress of the procedure ensured sensitivity and reduced the number of required SELEX rounds. Combining the enhanced procedure with high-throughput sequencing and intensive bioinformatics analysis guaranteed the identification of all possible aptamers in contrast to the conventional cloning and Sanger sequencing method. It also allowed the detection of cycle-by-cycle changes occurring throughout the SELEX procedure to provide a more insightful interpretation of data and a better understanding of the SELEX enrichment process. HTS results also revealed that we could have selected our specific aptamer after only two positive cycles. The most promising aptamer selected through our enhanced cell-SELEX method was BR8-15. It showed good binding affinity in the nanomolar range, high sensitivity (LOD = 10^1^ CFU/mL), and specificity towards the three classic *Brucella* species.

## Figures and Tables

**Table 1:** Selected aptamer sequences from the top ten enriched sequences showing their ranking and normalized frequency in BR8 and BR6.

Rank in BR8	Normalized Frequency	Name	Sequence	Rank in BR6 (Aptamers Bound to Non-Target Cells)/Normalized Frequency
#1	27371.16	BR8-1	TGTGGCAGACGGATGACCACGCAGGGTCGGTTGGCGAGAGTGGGTCTTAACTGGTGGGTTGCGGCTGGTTGGGAGGCTGTCATGGAGTGA	3/1446.38
#3	8497.90	BR8-3	TGTGGCAGACGGATGACACGATAAGTGGATCACGGTGTGCGAGGTCGGGGGGAGGGGTGCAGAAGTGGTCGTGAGGCTGTCATGGAGTGA	12/619.03
#15	3282.00	BR8-15	TGTGGCAGACGGATGACGCGGACCGGGATCGCTGTCCATGGGGTTAGAGCAGTGCGGTGGTGTTGTGCTGGTGAGGCTGTCATGGAGTGA	5145/11.90

**Table 2:** Illustration of selection pressure applied throughout the enhanced Cell-SELEX procedure for selecting an aptamer specific for the three classic *Brucella* species; *B. abortus*, *B. melitensis*, and *B. suis*, reported in Egypt.

Round No.	Cell-SELEX Type	Selection Pressure							
		BSA (mg/mL)	tRNA (mg/mL)	Bacterial Cell Concentration (CFU/mL)	Co-Incubation Time (min)	Shaking (rpm)	Washing Volume (µL)	Washing Frequency	Washing Incubation Time (min)
1	Conventional	1	0.1	10^8^	45	200	250	1	1
2	Negative	1	----------	10^8^	45	200	250	1	1
3	Toggle	20	0.2	10^7^	30	400	500	2	3
4	Negative	1	---------	10^7^	30	200	500	2	1
5	Conventional	40	0.3	10^6^	20	600	750	3	5
6	Conventional	60	0.4	10^5^	15	800	1000	3	5

## Data Availability

Not applicable.

## Supplementary Materials

The following supporting information can be downloaded at: https://www.mdpi.com/article/10.3390/ijms23116131/s1.
