# Supplementary material for: Generation and Selection of Specific Aptamers Targeting Brucella Species through an Enhanced Cell-SELEX Methodology
Source: Int J Mol Sci. 2022 May 30;23(11):6131. doi: 10.3390/ijms23116131 (PMC9180945; doi:10.3390/ijms23116131)
Supplement: Supplementary file 1 [file ijms-23-06131-s001.zip › ijms-1696036-supplementary.pdf]

Generation and selection of specific aptamers targeting Brucella species through an enhanced cell-SELEX methodology

Dalia M. El-Husseini [1,2,*], Ashraf E. Sayour [3], Falk Melzer [2,*], Magda F. Mohamed[4], Heinrich Neubauer [2] and Reham H. Tammam [4]

[1] Biotechnology Department, Animal Health Research Institute, Agricultural Research Center, Dokki, Giza 12618, Egypt
[2] Institute of Bacterial Infections and Zoonoses, Friedrich-Loeffler-Institut, 07743 Jena, Germany; Heinrich.Neubauer@fli.de
[3] Molecular Biomimetics Research Group, Animal Health Research Institute, Agricultural Research Center, Dokki, Giza 12618, Egypt; sayourashraf@gmail.com
[4] Chemistry Department, Faculty of Science, Cairo University, Giza 12613, Egypt; magdafikry85@yahoo.com, reham_tammam@cu.edu.eg
* Correspondence: dalia_biotech@yahoo.com (D.M.E.-H.); Falk.Melzer@fli.de (F.M.)

Content

1. PCR validation for amplification of aptamer sequences
   a. Asymmetric PCR
   b. Symmetric followed by Asymmetric PCR
2. Real-time PCR (qPCR) monitoring results
3. HTS result analysis workflow
4. Preparing aptamer pools for HTS sequencing
5. HTS result bioinformatics analysis
6. Specificity analysis for candidate aptamers
7. Binding affinity for BR8-15
8. Detection limit for BR8-15

1. PCR validation for amplification of aptamer sequences

   a. Asymmetric PCR

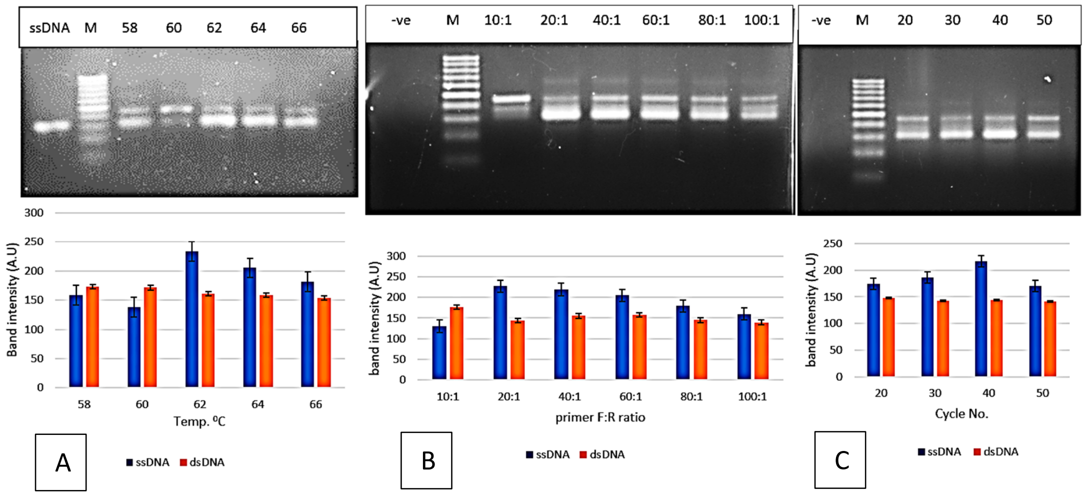

Figure S1 illustrate annealing temperature(A), primer ratio F:R (B) and cycle number(C) validation for direct asymmetric PCR

b. Symmetric then asymmetric PCR

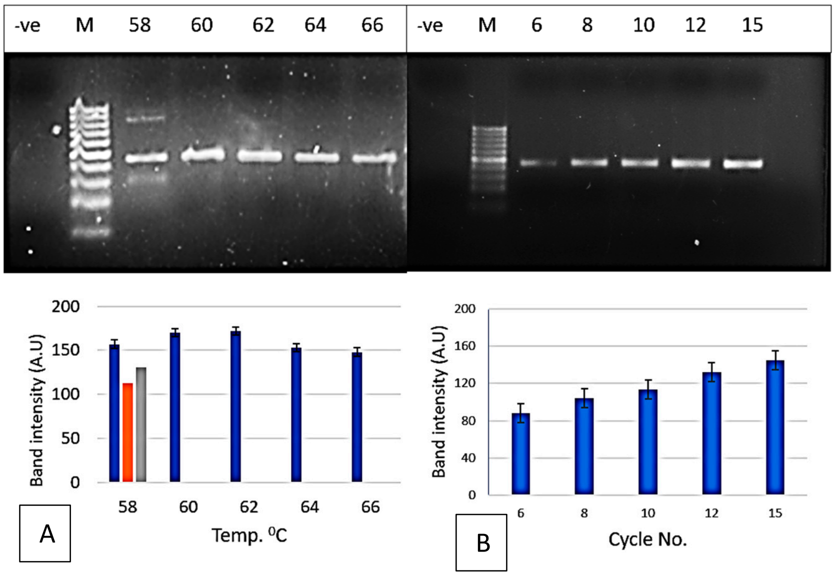

Figure S2 illustrate annealing temperature(A) and cycle number(B) validation for symmetric PCR

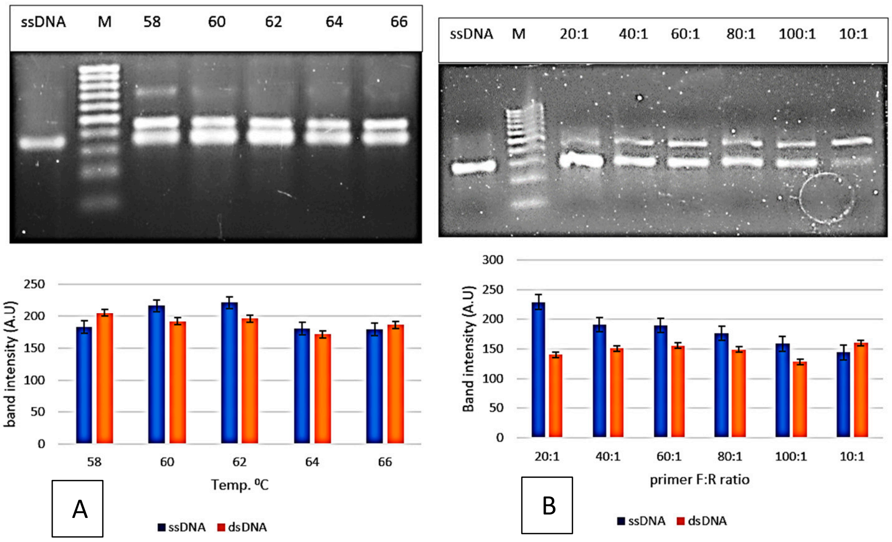

Figure S3 illustrate annealing temperature(A) and primer ratio F:R (B) validation for asymmetric PCR following the symmetric PCR.

2. Real-time PCR (qPCR) monitoring results

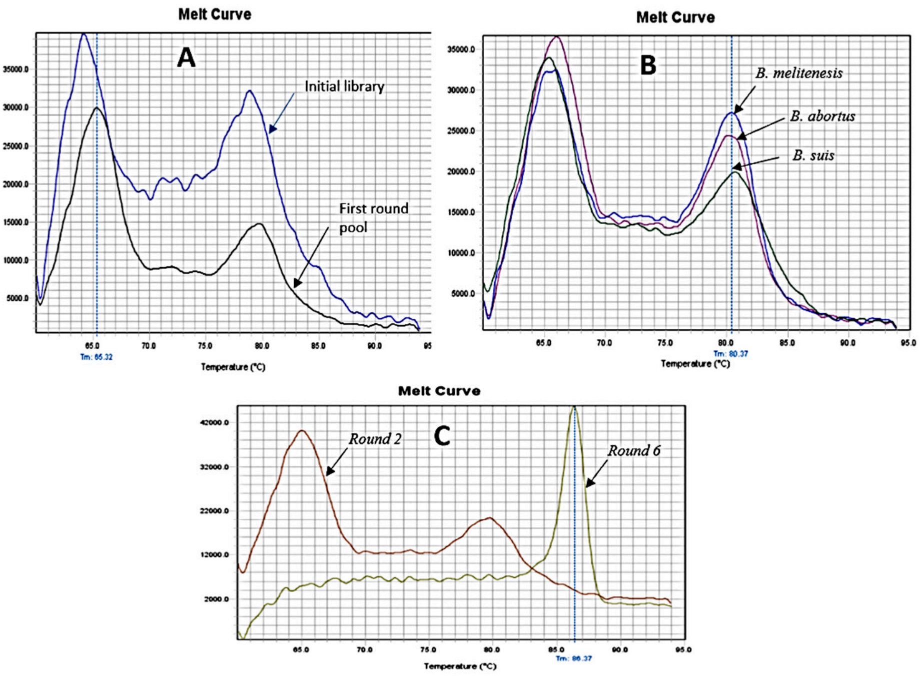

Figure S4 qPCR melting curves, A) initial library pool versus the produced aptamer pool from the cell-SELEX first round. B) the produced aptamer pool from toggle cell-SELEX of B. melitensis, B. abortus and B. suis. C) negative selection cycles (round 2 versus round 6)

3. HTS result analysis workflow

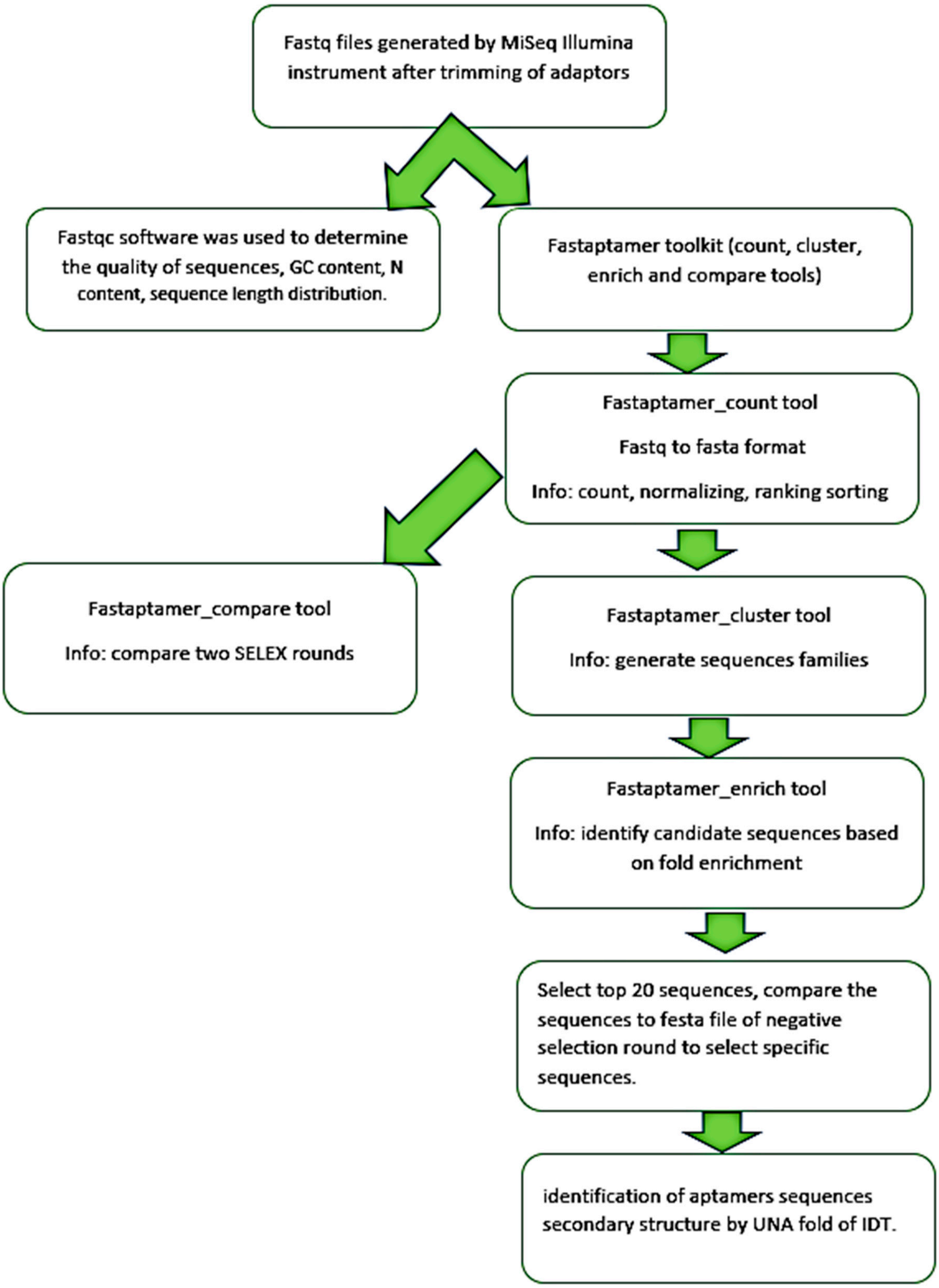

Figure S5 illustrates HTS results analysis workflow.

4. Preparing aptamer pools for HTS sequencing

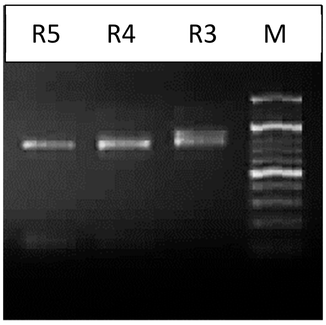

Figure S6 shows an example for aptamer pool preparation of rounds (3,4 and 5) for HTS sequencing by adding the overhang primers by PCR resulting in a band at 162 bp.

5. HTS result bioinformatics analysis

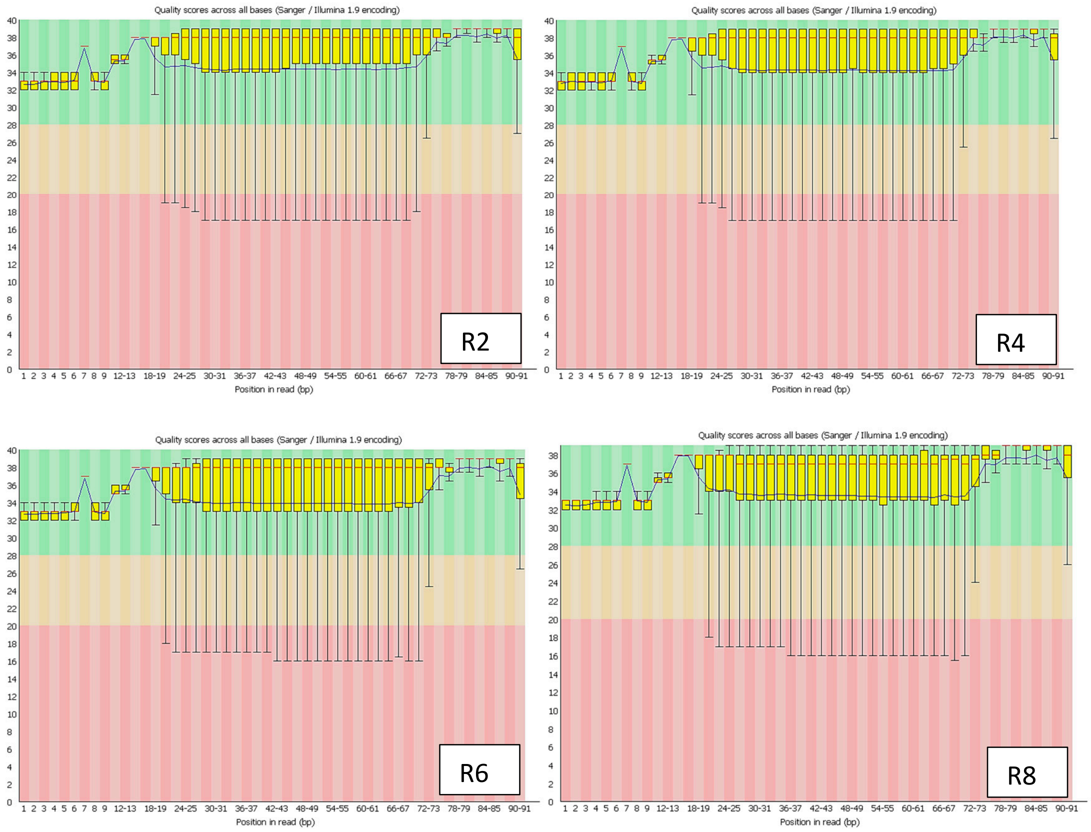

Figure S7 Illustrates the quality values across all bases at each position in the FASTQ file for some examples of cell-SELEX rounds no. 2,4,6,8. aptamer N sequence starts from base number 18 to 72. Before and after this base number are forward and reverse primers, respectively. each graph is divided into three quality ranges: good (green), reasonable (orange) and poor (red).

Table S1 shows GC percentage detected by fastQC analysis for each round. note that for round 2 and 6 (negative selection rounds), we analyzed the aptamer sequences present in the cell-SELEX reaction supernatant.

Round number	GC content by FastQC analysis
1	51%
2	54%
3	56%
4	56%
5	56%
6	57%
7	58%
8	59%

Table S2 Top 10 Sequences selected based on showing almost constant enrichment throughout all rounds and high enrichment through the last two positive selection rounds listed in order.

Sequence	Rank according to enrichment comparison through rounds
TGTGGCAGACGGATGACGCGGACCGGGATCGCTGTCCATGGGGTTAGAGCAGTGCGGTGGTGTTGTGCTGGTGAGGCTGTCATGGAGTGA	1
TGTGGCAGACGGATGACCACGCAGGGTCGGTTGGCGAGAGTGGGTCTTAACTGGTGGGTTGCGGCTGGTTGGGAGGCTGTCATGGAGTGA	2
TGTGGCAGACGGATGACACGATAAGTGGATCACGGTGTGCGAGGTCGGGGGGAGGGGTGCAGAAGTGGTCGTGAGGCTGTCATGGAGTGA	6
TGTGGCAGACGGATGACACGGACGGTGATAAGGTGTGGTTCCTGTGCTAGTGGTAGTTTGGGGTTTGGGGGGGAGGCTGTCATGGAGTGA	9
TGTGGCAGACGGATGACGCGGTGGGACGGGAGCAAATAGTATAACGGAGGACGGCGGTTTCTCGGTGGTGTGGAGGCTGTCATGGAGTGA	13
TGTGGCAGACGGATGACCACGAGAGGGTGCCGGTTCTGTCCTCGAAGGAGAAGCTGGTGTTCTGGCGGTGGTGAGGCTGTCATGGAGTGA	28
TGTGGCAGACGGATGACCACCGCGCGCTGGGGGTCCGCGGGCTAGCCGGGTGCAGGTCTGTTGGCTGGTGGGGAGGCTGTCATGGAGTGA	34
TGTGGCAGACGGATGACCGGCGCAGCAGGCGAGCTGGTGTGGTGGTGGGTTGGATGGTGAACATAGTGGTTGGAGGCTGTCATGGAGTGA	39
TGTGGCAGACGGATGACGGCAGCGGCTCAGAGATTGCAGTACCGAGGGGCGTCGCGGCTTTTGGTGGGGGTGGAGGCTGTCATGGAGTGA	47
TGTGGCAGACGGATGACCACGCGGAGGACGGTATGGAGATTTTGTCGGGCCGGGGTGTGCTGTTGACGGAGGGAGGCTGTCATGGAGTGA	48

Table S3 Example of sequence appeared in R4 and continued in low frequency till end of cell-SELEX (1), and sequence appeared in R5 and decreased in frequency in the following rounds (2).

No.	sequence	R1-R3	R4	R5	R6-R8
1	TGTGGCAGACGGATGACGGGGCTGCCGGAGGAGTGCCGTATCCC<br>AACAAGGTCTCCAGGGGTAATGGTGGTGGAGGCTGTCATGGAGTGA	0 reads	2 reads	26 reads	<100 reads
2	TGTGGCAGACGGATGACCACACATGGAAGGTTTCCGGGTATCTTGC<br>GCGATTCGGCATGTCGAGTGGAGGTGGAGGCTGTCATGGAGTGA	0 reads	0 reads	20 reads	<10 reads

6. Specificity analysis for candidate aptamers

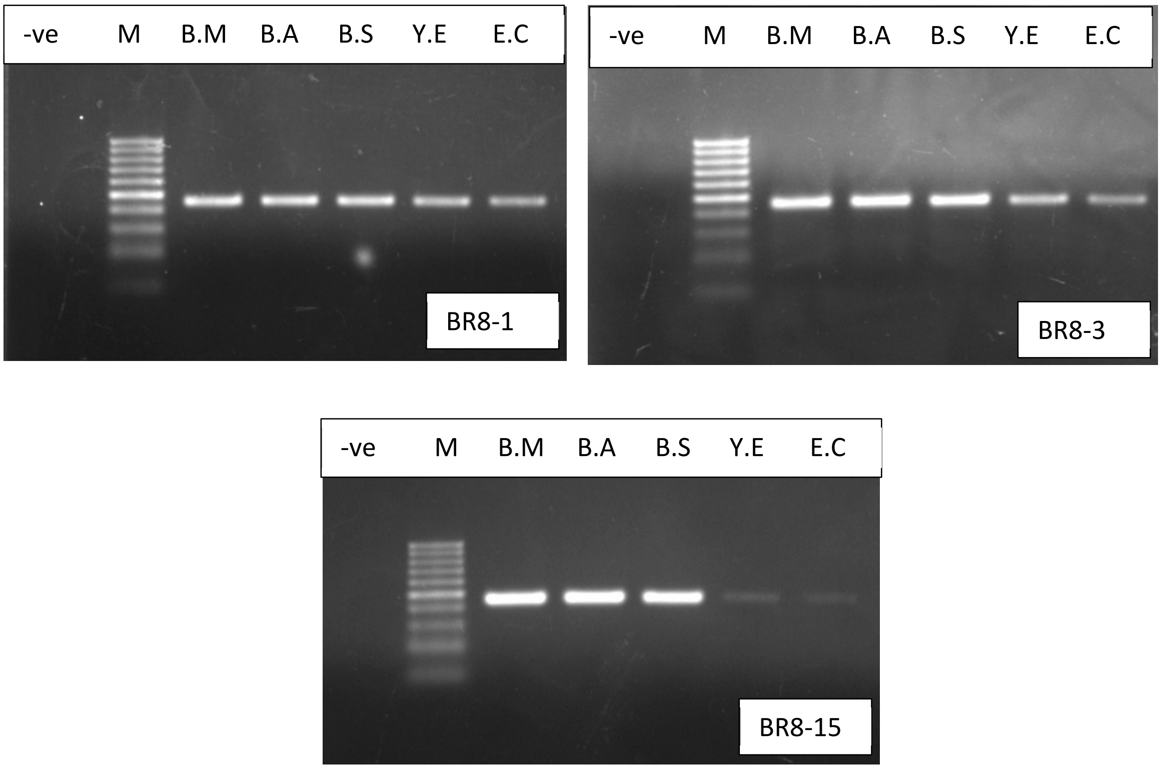

Figure S8 Gels specificity analysis for BR8-1, BR8-3 and BR8-15 aptamers.

7. Binding affinity for BR8-15

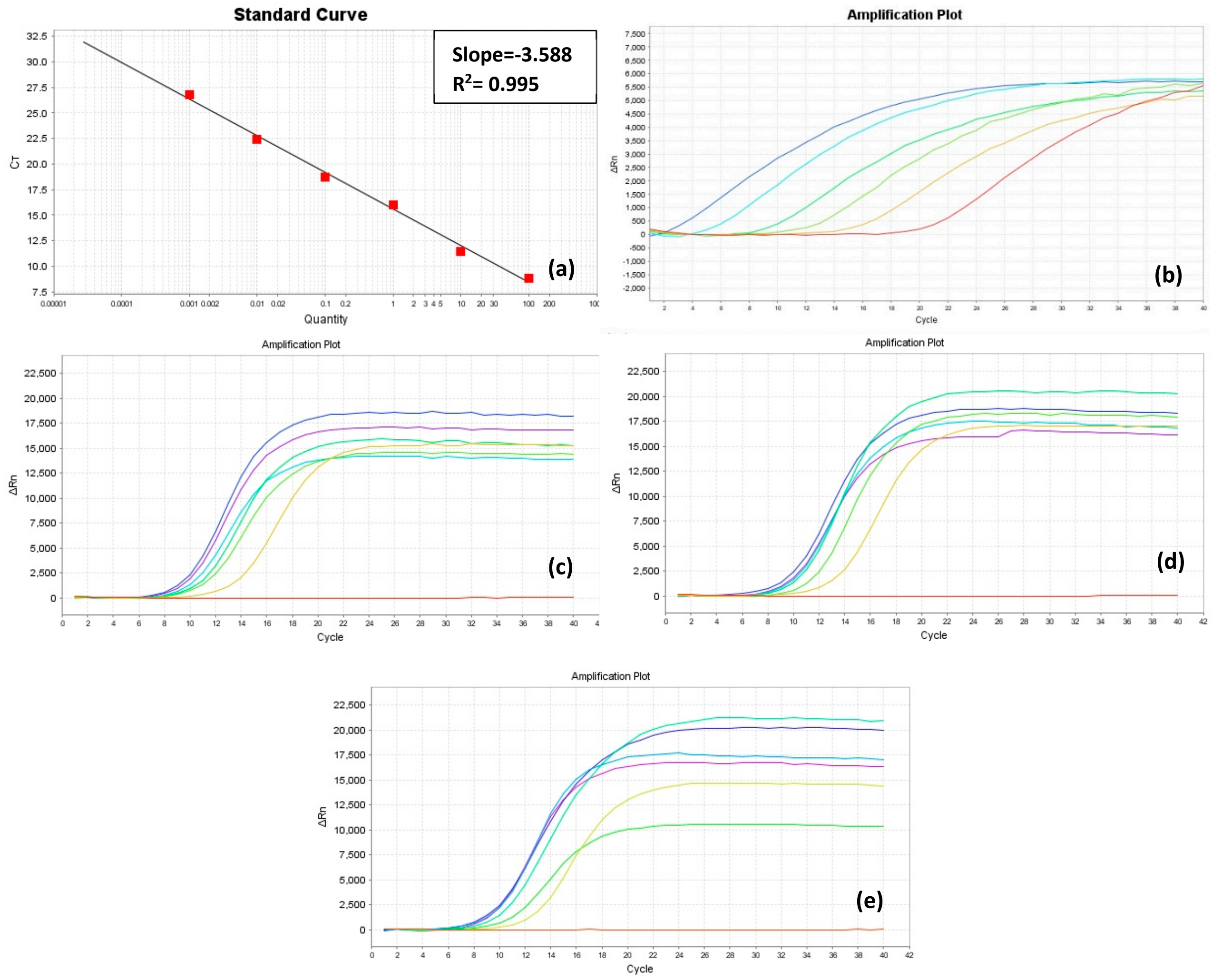

Figure S9 Standard curve constructed by varying the aptamer concentration from 10^{-3} to 10^{2} (a), and their amplification plot (b). Examples of amplification plots used for binding affinity analysis of BR8-15 towards B. melitensis (c), B. abortus (d) and B. suis (e).

8. Detection limit for BR8-15

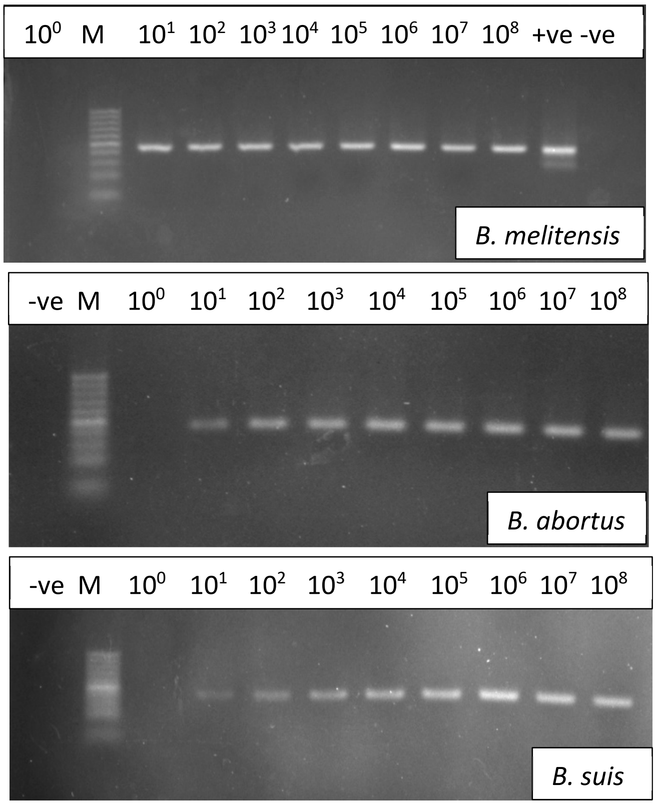

Figure S10 Gels for limit of detection determination of BR8-15 aptamers towards B. melitensis, B. abortus and B. suis.
